# Patterns of myocardial fibrosis by CMR in patients with conduction abnormalities

**DOI:** 10.1186/1532-429X-14-S1-P213

**Published:** 2012-02-01

**Authors:** Omar Cheema, Muhammed Umair Qamar, Jiaqiong Xu, Miguel Quinones, William A Zoghbi, Dipan J Shah

**Affiliations:** 1 Division of Cardiology, University of Texas Medical Branch, Galveston, TX, USA; 2 Cardiology, Methodist DeBakey Heart & Vascular Center, Houston, TX, USA

## Summary

Cine and DE-CMR performed in a cohort of patients revealed that the presence of LBBB was more frequently associated with increases in LV volumes and depression in LV function, whereas RBBB or nonspecific conduction delay were more strongly associated with septal replacement fibrosis.

## Background

Ventricular conduction delay is thought to occur from a variety of differing mechanism including ischemia or infarction, left ventricular (LV) hypertrophy and/or dilation, and fibrotic degenerative disease of the conduction system. CMR is an ideal modality to evaluate for these mechanisms as cine-CMR is the “gold-standard” for assessment of cardiac morphology and function, and delayed enhancement (DE)-CMR has emerged as a robust modality for assessment of LV replacement fibrosis.

## Objective

To compare the prevalence of abnormalities on cine and DE-CMR in patients with various forms of ventricular conduction delay on 12 lead ECG.

## Methods

We prospectively enrolled 698 consecutive patients presenting to our center from July, 2009 to March, 2010 for CMR for a variety of clinical indications. Patients were categorized based on QRS morphology on concurrent 12-lead ECG tracings into the following 4 groups: 1) no conduction delay (QRS duration ≤ 100 msec); 2) nonspecific conduction delay (NSCD) [QRS duration more than 100 but less than 120 msec]; 3) LBBB (QRS duration ≥ 120 msec with LBBB morphology by Minnesota code); and 4) right bundle branch block (RBBB) [QRS duration ≥ 120 with RBBB morphology by Minnesota code]. Cine and DE-CMR images were analyzed for left atrial (LA) volumes, LV and right ventricular (RV) volumes and ejection fraction (EF), LV mass, and LV replacement fibrosis per standard methodologies for our laboratory.

## Results

The baseline clinical characteristics of our cohort included cardiac risk factors of hypertension in 401 (57%); diabetes in 182 (25%); hyperlipidemia in 310 (44%); and tobacco abuse in 57 (8%). Symptoms of chest pain were present in 170 (25%) and dyspnea in 289 (41%). Prior history of myocardial infarction was present in 94 (13%). The systolic blood pressure was 127 mmHg ± 21; diastolic blood pressure was 72 mmHg ± 12; the body surface area was 1.94 kg/m^2^ ± 0.27; heart rate was 75 bpm ± 14; and LVEF 57% ± 17. Imaging characteristics as segregated based on the QRS complex are shown in Table 1. Note that patients with LBBB had increased LV volumes, and reduced LVEF compared to all other groups. Interestingly total LV fibrosis (as % of LV) or septal location fibrosis was not different between LBBB patients and the other groups. However patients with RBBB or NSCD had a greater proportion of septal fibrosis and the latter also had a greater total LV fibrosis extent.

**Table 1:** 

Variable	LBBB (n=22)	RBBB (n-40)	NSCD (n=113)	No Delay (n=523)
LA Volume (ml)	133 ± 53.5	118 ± 63.4	121 ± 46.3^a^	105 ± 43.7
LVEDV (ml)	231 ± 109^b,c,d^	139 ± 59^e^	179 ± 71	141 ± 60
LVESV (ml)	162 ± 97^b,c,d^	65 ± 47	93 ± 68^a^	64 ± 54
LV Myocardial Mass (g)	176 ± 75	154 ± 59	177 ± 65^a^	146 ± 60
LVEF (%)	35.4 ± 17.6^b,c,d^	56.7 ± 14.1	52.8 ± 19.8^a^	59.1 ± 16.8
RVEDV (ml)	130 ± 60	161 ± 79^f^	144 ± 53	130 ± 60
RVESV (ml)	75 ± 62	90 ± 62^f^	74 ± 45	63 ± 33
RVEF (%)	49.0 ± 18.4	47.1 ± 10.8	50.7 ± 13.8	53.0 ± 12.1
Scar Extent (% of LV)	4.3 ± 7.1	5.5 ± 8.7	7.7 ± 12.6^a^	4.1 ± 9.2
Septal Location Fibrosis	3 (13.6)	10 (25.0)^f^	31 (27.4)^a^	35 (6.7)

Data are presented as mean ± SD for continuous variables and number (%) for categorical variables. P-values are based on one-way analysis of variance (ANOVA) with Bonferroni correction for multiple pairwise comparisons for continuous variables and Fisher’s exact test for categorical variables. ^a^P<0.05 for comparison between NSCD and normal ^b^P<0.05 for comparison between LBBB and normal ^c^P<0.05 for comparison between LBBB and NSCD ^d^P<0.05 for comparison between LBBB and RBBB ^e^P<0.05 for comparison between RBBB and NSCD ^f^P<0.05 for comparison between RBBB and normal

## Conclusions

RBBB or NSCD are more strongly associated with septal replacement fibrosis whereas LBBB is more frequently associated with increases in LV volumes and depression in LV function.

## Funding

None.

